# Associations of limbic-affective brain activity and severity of ongoing chronic arthritis pain are explained by trait anxiety

**DOI:** 10.1016/j.nicl.2016.06.022

**Published:** 2016-07-01

**Authors:** William J. Cottam, Laura Condon, Hamza Alshuft, Diane Reckziegel, Dorothee P. Auer

**Affiliations:** a Arthritis Research UK Pain Centre, University of Nottingham, Nottingham, UK; b Sir Peter Mansfield Imaging Centre, University of Nottingham, Nottingham, UK; c Division of Clinical Neuroscience, Radiological Sciences, University of Nottingham, Queen's Medical Centre, Nottingham, UK

**Keywords:** Chronic pain, Knee osteoarthritis, Neuroimaging, Arterial spin labelling

## Abstract

Functional magnetic resonance imaging studies (fMRI) have transformed our understanding of central processing of evoked pain but the typically used block and event-related designs are not best suited to the study of ongoing pain. Here we used arterial spin labelling (ASL) for cerebral blood flow mapping to characterise the neural correlates of perceived intensity of osteoarthritis (OA) pain and its interrelation with negative affect. Twenty-six patients with painful knee OA and twenty-seven healthy controls underwent pain phenotyping and ASL MRI at 3T. Intensity of OA pain correlated positively with blood flow in the anterior mid-cingulate cortex (aMCC), subgenual cingulate cortex (sgACC), bilateral hippocampi, bilateral amygdala, left central operculum, mid-insula, putamen and the brainstem. Additional control for trait anxiety scores reduced the pain-CBF association to the aMCC, whilst pain catastrophizing scores only explained some of the limbic correlations. In conclusion, we found that neural correlates of reported intensity of ongoing chronic pain intensity mapped to limbic-affective circuits, and that the association pattern apart from aMCC was explained by trait anxiety thus highlighting the importance of aversiveness in the experience of clinical pain.

## Highlights

• The neural basis of chronic OA pain was investigated using 3D-ASL MRI.
• Reported OA pain intensity is associated with CBF in limbic and nociceptive regions.
• This association is strongly moderated by anxiety.
• These results highlight the role of limbic processing in ongoing chronic OA pain.

## 1 Introduction

Chronic pain affects approximately 11% of the population, with poor outcomes for current treatment (Harstall and O., 2003). Large surveys across Europe and Canada found that arthritis/osteoarthritis (OA) joint pain was the most common cause of chronic pain, reported by over one third of chronic pain patients (Breivik et al., 2006, Schopflocher et al., 2011). Pain is a primary symptom of OA, a degenerative joint disease, but there is disagreement on how well structural damage (as evidenced by radiographs) concurs with the severity or presence of symptoms including pain (Hannan et al., 2000). Previous studies of chronic OA pain have suggested that the pain experience is not only the result of constant or aberrant nociceptive drive due to joint tissue damage or inflammation (Mease et al., 2011) but is also inclusive of psychological factors such as anxiety and depression (Marks, 2009, Axford et al., 2010, Edwards et al., 2011). Neuroimaging studies have found functional and structural brain changes in chronic pain patients thought to reflect brain plasticity and potentially providing targets for pharmacological and psychological therapies (Davis and Moayedi, 2013).

Despite the increasing interest in neuroimaging studies in chronic pain, findings are often inconsistent not only between different pain aetiologies but also in chronic musculoskeletal pain. The brain response to evoked pain was abnormal in some studies in patients with OA especially in those reporting hyperalgesia, with other studies of OA and chronic lower back pain subjects not reporting differences from controls (Gwilym et al., 2009, Apkarian et al., 2005, Parks et al., 2011, Sofat et al., 2013, Wasan et al., 2011, Hiramatsu et al., 2014). Similar discrepancies between studies were shown for other chronic pain cohorts such as fibromyalgia and chronic regional pain syndrome (Freund et al., 2011, Kamping et al., 2013, Lebel et al., 2008, Pujol et al., 2009). These discrepancies might have arisen from the challenge to induce comparable pain states between patients and controls when using fixed stimulus intensity (Ducreux et al., 2006, Gwilym et al., 2009) rather than comparable perceived pain intensity (Hiramatsu et al., 2014, Gracely et al., 2002).

Moreover, experimentally evoked pain is unlikely to reproduce the full subjective experience of chronic pain with its aversive nature related to individual fears, beliefs and memories. To overcome these limitations, it would be desirable to directly study ongoing arthritis pain with appropriate methods that allow assessment of particular brain states. One such method is positron emission tomography (PET) using radioactive tracers to map cerebral glucose metabolism which revealed marked differences in the glucose metabolic pattern during clinical arthritis pain compared with experimental pain (Kulkarni et al., 2007). Alternatively, MRI based mapping of cerebral blood flow (CBF) using an arterial magnetic spin label (ASL) has shown promise to noninvasively study pain states in post-surgical pain, fibromyalgia, post-herpetic neuralgia, chronic low back pain and OA patients (Howard et al., 2011, Howard et al., 2012, Liu et al., 2013, Wasan et al., 2011, Shokouhi et al., 2015). Whole-brain ASL was recently used for covariance analysis of CBF maps with perceived intensity of capsaicin-induced pain in healthy controls (Segerdahl et al., 2015). The experimental approach also differs from earlier BOLD fMRI studies based on correlating perceived pain intensity across stimulation blocks (Boly et al., 2007, Christmann et al., 2007, Moulton et al., 2012, Peyron et al., 2007, Straube et al., 2009) by using an acute noxious stimulus to induce a prolonged pain state similar to Favilla et al. (2014). This whole-brain ASL correlational approach seems ideally suited to investigate clinical ongoing pain, thereby overcoming the experimental challenge to induce clinically relevant pain and the need for defining *a priori* regions of interest (Howard et al., 2012).

There is accumulating evidence linking the presence of chronic pain to increased levels of negative affect, including anxiety and depression (Axford et al., 2010, Marks, 2009), with some theorising that chronic pain and negative mood together form a continuum of aversive learning (Baliki and Apkarian, 2015). It is however unclear how this increase in negative affect relates to changes in brain function in chronic pain, as investigation of their interrelation has not been systematic.

Against this background, we aimed to use ASL to identify and characterise the neural correlates of clinical knee OA pain. Specifically, we hypothesised that brain areas encoding ongoing pain intensity overlap with limbic networks, and that the co-activation pattern can be partly explained by negative affect.

To test these hypotheses, we investigated the covariance pattern of regional CBF, indexing neural activity, with subjective rating of ongoing pain in chronic knee OA patients. We then repeated partial correlation analysis controlling for markers of negative affect that showed associations with pain severity.

## 2 Material and methods

### 2.1 Subjects and materials

Ethical approval was granted by Nottingham Research Ethics Committee 2 (Ref: 10/H0408/115). A total of 43 patients (median age 67.0 years, range 45–84 years, range of pain duration 12–456 months, 19 males) with radiographically defined unilateral chronic knee osteoarthritis and 30 healthy controls (median age 64.5 years, age range 43–80 years, 11 males) were included after giving written informed consent. Imaging data was excluded if of poor quality due to movement or imaging artefacts (patients = 8, controls = 3) and also patients reporting no pain on the day were excluded (n = 9). Group demographics after exclusions can be found in Table 1.

Directly before the scan session, all subjects underwent questionnaire assessments studying levels of education (where a score of 1 represents the attainment of a higher degree and 8 represents no educational attainment, adapted from Egerton and Mullan (2008)), pain severity (Visual Analogue Scale, VAS; 0–100), anxiety (State-Trait Anxiety Inventory, STAI), neuropathic-like pain components (PainDETECT – only in the patient cohort), pain catastrophizing (Pain Catastrophizing Scale, PCS) and depression (Beck's Depression Index, BDI-II) (Beck et al., 1996, Spielberger et al., 1983, Freynhagen et al., 2006, Sullivan et al., 1995). As BDI-II and PainDETECT scores show non-parametric properties, these scores were converted following Rasch analysis to allow use in linear analyses (unpublished data; see supplementary material for full details).

#### 2.1.1 MRI data acquisition

Subjects underwent multimodal MRI at 3T (MR750 Discovery, GE Healthcare) using a 32-channel head coil. Only ASL data is reported alongside high-resolution T1-weighted, 3D-FSPGR scan of the whole-brain, used for registration (Flip angle = 12°, echo time [TE] = 3.172 ms, repetition time [TR] = 8.148 ms, inversion time [TI] = 450 ms, field of view [FOV] = 256 mm, slice thickness = 1 mm, matrix = 256 × 256). The ASL sequence combines pulsed-continuous ASL (pCASL) labelling with a 3D spiral read-out (Flip angle = 111°, TE = 10.5 ms, TR = 4632 ms, labelling duration = 1450 ms, post-labelling duration = 1525 ms, FOV = 240 mm, slice thickness = 4 mm, slice gap = 4 mm, number of slices = 36, echo train length = 1, number of excitations = 3, matrix = 128 × 128) (Dai et al., 2008). Background suppression was used and an M_0_ image collected for image quantification. T1-weighted images were acquired parallel to the AC-PC line whilst the bottom of the acquired ASL image was positioned just below the cerebellum to allow whole-brain CBF imaging.

### 2.2 Image processing

Cerebral blood flow (CBF) maps (ml/100 g/min) were generated using an automatic reconstruction script as reported in Zaharchuk et al. (2010). The data was then manually brain-extracted using NeuRoi (http://www.nottingham.ac.uk/scs/divisions/clinicalneurology/software/neuroi.aspx), registered linearly (12 DOF) to MNI-space with FSL-FLIRT v6.0 (FMRIB software library) (Jenkinson et al., 2002) and smoothed to 8 mm FWHM in SPM8 (http://www.fil.ion.ucl.ac.uk/spm). In this study we were focussed only upon grey matter CBF linked to pain perception and hence used a grey matter mask to mitigate the multiple-test correction. For whole grey matter analyses, we used a dual-tissue probability mask (excluding ≤ 20% grey matter and ≥ 30% cerebrospinal fluid) based on the modified International Consortium for Brain Mapping (ICBM) tissue-probability maps provided in SPM8 (Rex et al., 2003). Probability thresholds were visually adapted to the 3D ASL dataset to increase grey matter specificity.

### 2.3 Statistical analyses

To address the main study aim we undertook a whole-brain grey matter correlation with reported VAS scores in OA subjects. Secondary tests included a between group comparison (all OA *vs*. HC), a subgroup comparison of those patients with left- or right-lateralised knee OA, and repeat correlation analyses with pain intensity 1) using data flipped in the x-axis (only data from participants with OA in the left knee were flipped), 2) controlling for any affective scores that correlated with reported pain intensities. All whole grey matter tests were corrected for age and sex, as well as for mean global CBF to control for inter-subject CBF differences of no interest using a GLM approach. Voxel-wise non-parametric permutation testing was carried out using FSL-randomise to correct for multiple comparisons (5000 permutations) and significance was defined as P < 0.05 FWE-corrected using threshold-free cluster enhancement (Nichols and Holmes, 2002, Smith and Nichols, 2009). In line with our hypothesis of a heightened limbic-affective component of clinical pain, we have extracted CBF values from those limbic/paralimbic regions of interest (ROI) that had shown significant CBF-VAS relationships in the initial regression analysis for selected *post*-*hoc* analysis to investigate interrelations with scores of negative affect (Rasch converted BDI, Trait anxiety and PCS scores). All psychometric scores of negative affect that presented either significant or trend correlations (P < 0.1) with VAS and limbic regional CBF were then taken forward for mediation analysis using the Process macros for SPSS (Hayes, 2015). In each case, reported VAS and regional CBF values were used as the independent (X) and dependent (Y) variables whilst psychometric scores were included as the mediating variable (M). Total and indirect effects of X on Y through M were assessed using 5000 bootstrap samples (bias corrected) and were considered significant if the 95% confidence interval did not include zero.

All demographic variables were compared between groups using an independent samples *t*-test. All tests were performed using SPSS 22 (IBM).

## 3 Results

### 3.1 General findings

No significant group differences were found in age or gender, but patients had lower educational levels. Self-reported mood was significantly lower in patients compared with controls, but not fulfilling of clinical depression criteria, as maximum scores classified only as ‘mild’ low mood (Beck et al., 1996). Patients also scored significantly higher on state and trait anxiety scales but not pain catastrophizing. Results from independent *t*-tests can be found in Table 1.

Global grey matter CBF did not differ (P = 0.52, age and sex corrected) between OA (44.0 ± 7.8 ml/100 g/min) and HC (45.8 ± 10.5 ml/100 g/min). Neither educational levels, reported VAS, BDI, PCS, nor STAI scores significantly correlated with global grey matter CBF values (Educational, P = 0.809; VAS, P = 0.573; BDI, P = 0.203; PCS, P = 0.449; State anxiety, P = 0.987; Trait anxiety, P = 0.639; controlled for age and sex). Trait anxiety and pain catastrophizing scores were found to significantly correlate with reported VAS (Trait anxiety – R = 0.643, P < 0.001; PCS – R = 0.564, P = 0.003) whilst Rasch converted BDI and state anxiety scores did not (BDI - R = 0.116, P = 0.573; State anxiety – R = 0.333, P = 0.104).

### 3.2 Association of local CBF with ongoing pain intensity in OA patients

**Fig. 1:**
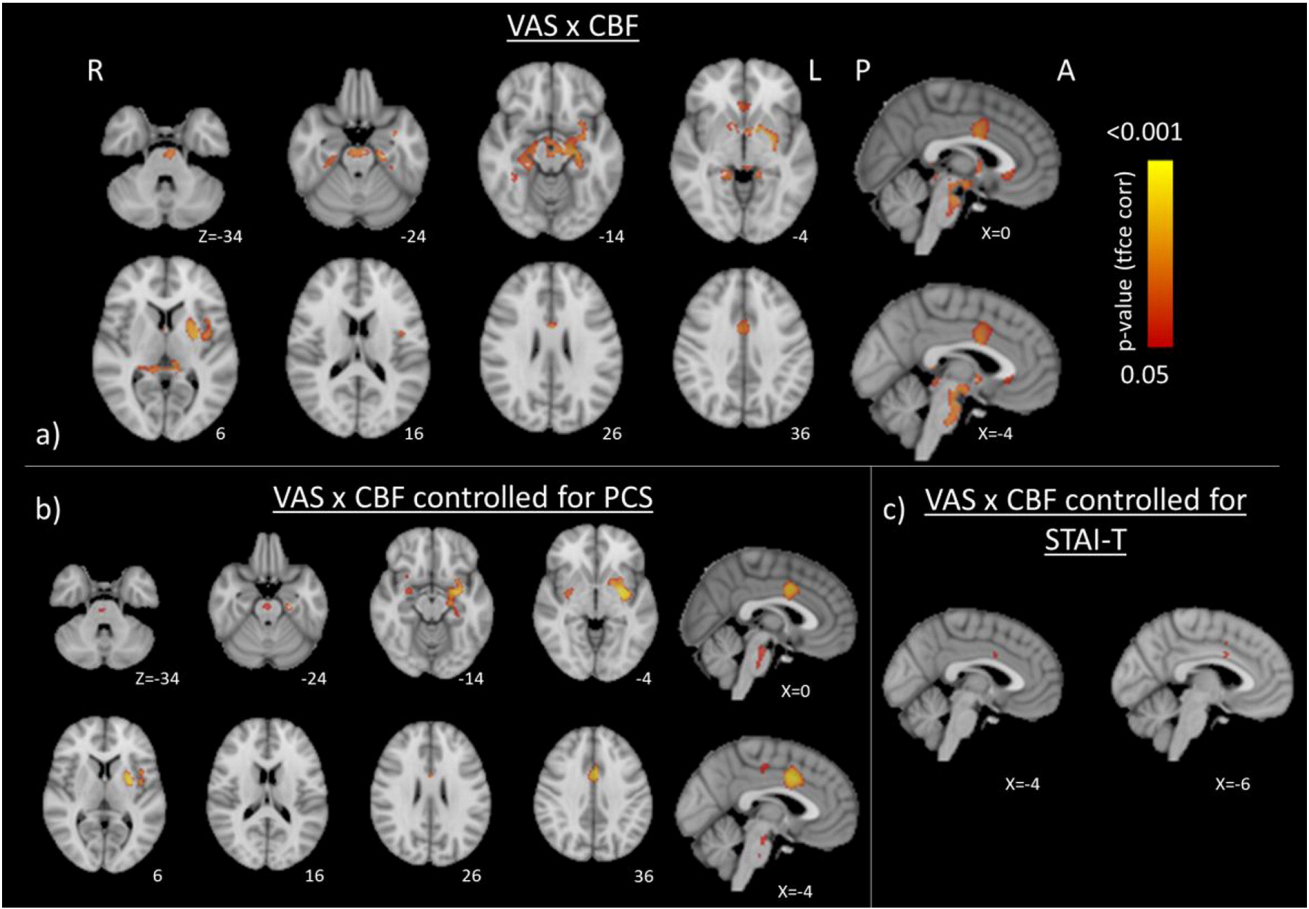
Brain regions where CBF correlates positively (FWE P < 0.05) with reported VAS scores after a) correcting for age, sex, and mean CBF and additionally for b) pain catastrophizing or c) trait anxiety scores. All images are shown in radiological format (right hemisphere is displayed on the left of the figure).

**Fig. 2:**
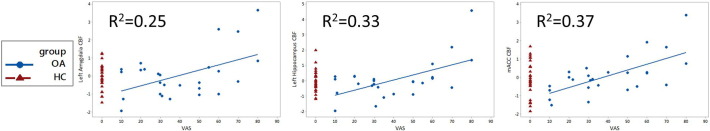
Scatter plots displaying partial correlations of reported pain intensity (VAS) and cerebral blood flow in healthy controls (HC; red) and chronic OA pain patients (OA; blue) in the left amygdala, hippocampus and the anterior mid-cingulate cortex (aMCC) controlled for age, sex and mean grey matter CBF.

Using voxel-wise correlation analysis grey matter CBF was positively correlated with reported VAS scores in few areas previously reported to be active during experimentally induced nociception (Wager et al., 2013), mainly the anterior mid-cingulate (aMCC). Importantly, we found additional positive covariance of CBF with VAS scores in emotional, fear and limbic areas, predominantly in the subgenual ACC (sgACC), bilateral hippocampi, left putamen and the bilateral amygdala (Fig. 1, Table 2). Illustrative scatter plots of this VAS × rCBF relationship are reported in Fig. 2.

As positive correlations were mainly lateralised in the left hemisphere, a Kruskal-Wallis *t*-test was used to investigate a potential bias from different pain intensity scores between subjects with left knee pain (n = 12) and those with right knee pain (n = 14). VAS scores were shown to not differ according to the laterality of knee pain (P = 0.411; controlled for age and sex). Whole-brain comparisons of left- and right-lateralised knee OA patients were also non-significant when corrected for multiple-comparisons (FWE, P > 0.05).

Re-running the main correlation analysis using data that had been flipped in the x-axis (only data from participants with OA in the left knee were flipped) to investigate if any regions displayed clear lateralisation. The results were centred on the midline with clusters once again found in the mACC, sgACC, left hippocampus and the right hippocampal/amygdala complex (FWE, P < 0.05). Two small clusters remained around the left putamen but there were no longer significant results within the left insula (Supplementary Fig. 2).

Due to PCS and trait anxiety scores displaying significant correlation with reported pain scores, we repeated the whole-brain correlation analysis whilst additionally controlling for trait anxiety and PCS scores (n = 25, as one subject was missing questionnaire data) to assess for putative confounding or mediating covariance with CBF. Controlling for trait anxiety resulted in markedly reduced associations, with only 2 clusters in the aMCC remaining significant (Fig. 1c, Table 3). Regressing out PCS scores, however, retained the correlations within the bilateral amygdala, left hippocampus, left putamen, left operculum and the aMCC, whilst correlations in the sgACC became non-significant. Additional clusters were also revealed in the posterior cingulate gyrus, right central operculum and the right putamen (Fig. 1b, Table 3).

#### 3.2.1 Interrelations with psychometrics of negative affect

To further characterise possible interrelations or putative mediation of CBF-VAS associations with negative affect, we performed *post hoc* tests on limbic regions where CBF was shown to significantly covary with VAS. As summarised previously, only PCS and trait anxiety were found to be significantly associated with severity of reported ongoing pain. No significant interrelations between CBF and psychometric scores were shown, with borderline associations noted between PCS and sgACC CBF (P < 0.1) and between BDI and bilateral amygdala CBF (P < 01, Table 4). Hence, PCS was further assessed for mediation effect, which was non-significant (95% confidence interval lower limit − 0.0596, upper limit 0.1946).

### 3.3 Group-wise whole-brain CBF comparison

Local CBF was not found to be significantly different in patients and controls (FWE, P > 0.05). There was also no difference after controlling for those psychometric scores showing between group significant differences (BDI, and trait anxiety scores). Single-group visualisations of CBF are provided in the Supplementary materials (Supp. Fig. 1).

## 4 Discussion

We studied the neural correlates of ongoing pain intensity in persistent knee OA pain using whole-brain CBF mapping, and present evidence for limbic, paralimbic and subcortical networks underpinning chronic pain perception in line with a strong affective, largely aversive dimension of the clinical pain experience. We also show that covariation of CBF and VAS in OA pain in the limbic and paralimbic regions was strongly modulated by trait anxiety.

We found that perceived intensity of OA pain in subjects with chronic painful knee OA was associated with a pattern of increased CBF involving nociceptive (anterior MCC, aMCC), (para-)limbic (bilateral amygdala, subgenual ACC, and hippocampus) networks and subcortical regions (the left putamen and brainstem). The aMCC has been consistently reported to underpin perceived pain intensity in both clinical OA patients and across previous studies in healthy controls (Boly et al., 2007, Christmann et al., 2007, Coghill et al., 1999, Favilla et al., 2014, Peyron et al., 2007, Straube et al., 2009). Animal research has shown that this cingulate sub-region contains a large cluster of nociceptive neurons (Lenz et al., 1998, Sikes and Vogt, 1992, Vogt, 2005). The aMCC was also the only region significantly covarying with perceived pain intensity after control of either trait anxiety or pain catastrophizing. This is in good agreement with the aMCC's reported role in pain processing, its consistent activation in nociceptive neuroimaging studies in control and clinical cohorts (Apkarian et al., 2005, Peyron et al., 2000, Tanasescu et al., 2016), and the proposition that it forms part of the neural signature of pain (Wager et al., 2013). This correlation also links to parallel work from our group finding a negative relationship between reported OA pain intensity and GABA levels in the aMCC, potentially alluding to the molecular mechanisms underlying chronic pain in this cohort (Reckziegel et al., 2016). Our partial correlation maps controlling for trait anxiety and trait catastrophizing show that the aMCC pain association is independent of negative affective trait and thus provide further support for the notion of a mainly nociceptive role of the aMCC activation in both experimental and clinical pain.

Apart from the common pain encoding role of the aMCC, there was a double dissociation between pain intensity encoding areas in patients and that reported previously in healthy controls. We did not find an association between activity in S1, S2, thalamus and bilateral anterior insula, brain regions that were previously reported to show covariance of perceived pain intensity and neural activity in experimental pain (Baliki et al., 2006, Boly et al., 2007, Christmann et al., 2007, Coghill et al., 1999, Moulton et al., 2012, Peyron et al., 2007, Roy et al., 2009, Straube et al., 2009, Favilla et al., 2014). Stimulus intensity as opposed to perceived pain intensity also correlated positively with activity in the bilateral insula and thalami in addition to S2 and aMCC (Atlas et al., 2014, Bornhovd et al., 2002, Lin et al., 2013, Oertel et al., 2012). Very few experimental nociceptive studies identified activity changes in the hippocampus or amygdala, but, those that do report amygdala activity give conflicting results (Atlas et al., 2014, Oertel et al., 2012, Peyron et al., 2007). No study reported activity in the sgACC in regards to pain- or stimulus-related intensity processing. Most of these investigations used BOLD fMRI (one used H_2_^15^O PET) as opposed to ASL which might have contributed to the observed differences. Nevertheless ASL measures CBF thus probing the same neurovascular coupling as BOLD, and is based on a direct physiological meaningful metric, identical to H_2_^15^O PET, supporting that the differences are due to chronic pain.

Whole-brain CBF covariance mapping revealed a unique subset of activity linked with reported ongoing pain intensity in the sgACC, bilateral hippocampi, left putamen and amygdala in chronic knee OA pain patients. None of these regions were noted to consistently encode pain intensity in healthy control studies (Apkarian et al., 2005, Duerden and Albanese, 2013). The reported covariation within the sgACC is intriguing as this region has not only been reported in other studies of ongoing pain in OA and chronic back pain (Baliki et al., 2008, Howard et al., 2012, Kulkarni et al., 2007, Parks et al., 2011), but is known to play a key role in affective disorders, with atrophy and functional hyperactivity reported in depressed individuals when compared to healthy subjects (Niida et al., 2014, Rodriguez-Cano et al., 2014, Connolly et al., 2013, de Kwaasteniet et al., 2013). By regressing out putative confounding effects from negative affect, we found that covariation of the clinical pain experience with sgACC CBF is explained by either pain catastrophizing or trait anxiety. Furthermore, sgACC CBF showed a borderline association with pain catastrophizing that was moderately correlated with VAS. However, *post hoc* analysis did not support a mediation effect.

In this study we found that bilateral hippocampal CBF significantly covaried with reported pain intensity. The left hippocampal CBF-VAS association was independent of catastrophizing whilst the right hippocampus became non-significant when controlling for catastrophizing scores. The hippocampus is a region typically known for the role it plays in memory and the limbic system, but it has also been observed to develop significant molecular changes such as decreased plasticity and neurogenesis in animal models of chronic neuropathic pain (Terada et al., 2008, Mutso et al., 2012). Human imaging studies have also reported increased hippocampal activity during ongoing OA pain (Howard et al., 2012, Parks et al., 2011) and in chronic back pain patients when compared with controls (Mutso et al., 2014). These results show that the hippocampus is involved in chronic pain and whilst the current study relates it to ongoing pain, previous studies of chronic pain have displayed evidence of hippocampal plasticity such as connectivity changes during the transition from subacute to persistent back pain (Mutso et al., 2014) and increased grey matter after an 11-week cognitive behavioural therapy intervention (Seminowicz et al., 2013).

Chronic pain has long been linked to increased distress and emotional aversiveness and the amygdala has been noted as an important region involved in the interaction between nociception and emotion (Neugebauer et al., 2004). Pre-clinical studies have, for example, shown evidence that lesioning of the amygdala in a neuropathic pain model “significantly inhibited the persistence of pain” (Li et al., 2013), and that application of CRF to the amygdala can trigger pain-related behaviour in the absence of any pain-related pathology or disease (Ji et al., 2013). Human neuroimaging studies also provide evidence that the amygdala is involved in the perception of chronic pain (Neugebauer et al., 2004, Hashmi et al., 2013, Liu et al., 2013). The covariance of both the amygdala and sgACC is furthermore in line with known functional and structural connectedness (Kellermann et al., 2013, Robinson et al., 2010, Simons et al., 2014), and concords with observations of increased metabolism within the left amygdala and sgACC in OA patients comparing high with low pain states or with pain free control subjects (Howard et al., 2012, Kulkarni et al., 2007, Parks et al., 2011). Importantly, a longitudinal fMRI study of back pain subjects demonstrated a shift of pain processing away from classical nociceptive areas towards emotion-related circuitry including the amygdala in patients who progressed from acute to persistent back pain (Hashmi et al., 2013). Our finding of pain intensity encoding for chronic pain in the amygdala is thus consistent with common reporting in chronic pain cohorts and development over pain chronification, and strengthens the notion of amygdala dysfunction as a mechanism contributing to persistent clinical pain (Hashmi et al., 2013, Howard et al., 2012, Kulkarni et al., 2007, Liu et al., 2013, Parks et al., 2011). Putaminal activation has previously been reported in ASL studies of post-surgical tooth pain, fibromyalgia, postherpetic neuralgia and subcutaneous evoked pain (Howard et al., 2011, Liu et al., 2013, Owen et al., 2012, Shokouhi et al., 2015). There is some suggestion of enlarged putaminal volumes in chronic pain cohorts (Desouza et al., 2013, Schmidt-Wilcke et al., 2006) and the putamen is structurally connected with a network of nociceptive, attentional and sensory-motor regions supporting a role of the putamen in the perception of pain (Starr et al., 2011). Interestingly, a previous study of catastrophizing in fibromyalgia patients reported greater putaminal and ACC activity in high catastrophizers (compared with low catastrophizers) whilst we find increased correlation in these regions even when controlling for PCS (Gracely et al., 2004). A plausible role of the putamen reported here with the amygdala-hippocampal complex and sgACC is in the learning and memory of past aversive pain experiences (Gramsch et al., 2014, Kattoor et al., 2013, Vogt, 2005). A putative link to acquired, learnt pain sensitivity would also be in line with the noted independence of the putaminal pain encoding from negative affective scores. Thus, the observed putaminal activation in ongoing clinical pain might be attributed to a conditioned aversive learned response to the subjective, and repeated, experience of chronic pain.

There is strong evidence reporting a relationship between chronic pain and anxiety. In line with previous findings, we found higher levels of trait anxiety in patients with chronic OA pain, and that anxiety scores were positively related with reported pain intensity (Axford et al., 2010, Marks, 2009). Trait anxiety scores were also correlated with pain catastrophizing scores. Importantly, trait anxiety scores accounted for most of the observed CBF-pain intensity correlations except within the aMCC suggesting that the involvement of the limbic-affective circuits in perceived clinical pain intensity is largely driven by negative affect and specifically anxiety. To our knowledge, previous ASL and fMRI studies in chronic pain patients did not control for trait anxiety. It is noteworthy that also the amygdala activation- pain relationship was also lost when covarying for trait anxiety, but not when controlling for pain catastrophizing. This suggests that the amygdala activation underpinning the severity of the clinical pain experience may reflect the predisposition to heightened anxiety. Whilst we did not find a mediation effect, the modulatory role of trait anxiety on amygdala's function in pain encoding could explain the inconsistent reports of amygdala activation in pain studies, and aligns well with the threat detection function of the amygdala. Moreover, the prominent role of trait anxiety for the neural underpinning of clinical pain converges with novel treatment concepts. Anxiety relieving interventions such as mindfulness-based training or prescription of duloxetine (mixed anxiolytic antidepressant profile) have been shown to reduce levels of chronic pain (Chappell et al., 2009, Pergolizzi et al., 2013, Rod, 2015).

Contradictory to previous studies of OA subjects, we found no significant differences in neural activity at rest between patients and controls even in the presence of ongoing mild pain (Gwilym et al., 2009, Howard et al., 2012). Results of no difference have also been reported in OA patients undergoing noxious stimulation and fibromyalgia patients at rest when compared to healthy controls (Kulkarni et al., 2007, Shokouhi et al., 2015, Parks et al., 2011). A potential explanation is that none of the regions we identified are specific to pain and as such, may serve multiple other functions (such as arousal, pain-unrelated discomfort) in the healthy subjects at the time of scanning which may have confounded our comparison. Additionally, the lack of independent association between limbic CBF and pain scores when accounting for trait anxiety, suggests that previous group differences might have been contributed to by an even greater group difference in negative affect than seen in our cohort. Moreover, our study is likely to have relatively low sensitivity to detect group differences in CBF as our cohort reported only mild-moderate ongoing pain levels and mild levels of anxiety and were well prepared to reduce inconvenience for the scanning sessions thus reducing potential differences compared to a control group. This does however not invalidate the findings from the more powerful within-group correlation analysis.

In this study, we combined an optimised MRI technique in a reasonably large, psychometrically well characterised patient sample with a novel voxel-based pain perception driven analysis to establish brain regions directly related to the perceived intensity of ongoing pain and for the first time investigated their interdependence from negative affective scores. ASL imaging affords several advantages in this regard over the blood-oxygen-level dependent (BOLD) fMRI by providing absolute measures of CBF (ml/100 g/min) as opposed to relative and physiologically compounded indices of blood oxygenation. ASL imaging displays enhanced sensitivity to tonic stimuli in comparison to BOLD which is ideal to investigate chronic pain states such as OA due to the tonic nature of chronic pain. This paradigm also minimises attentional confounds compared to fMRI studies looking at spontaneously fluctuating pain intensity that require continuous recording of pain ratings during the scan period.

The study had a number of limitations. In order to overcome the low signal-to-noise ratio, (SNR) inherent with ASL imaging, a 3D pCASL sequence with background suppression which has been shown to markedly improve the SNR (Dai et al., 2008) was used that is however sensitive to motion artefacts and did not allow to record pain scores concurrently. Secondly, the study is limited by its cross-sectional design thus only allowing observational findings. Thirdly, VAS scores were only recorded prior to the scan and no recordings were taken after to confirm that pain persisted during the scan. Lastly, without a larger sample size, the secondary tests assessing the effect of PCS and STAI scores on the VAS × CBF correlation are to be considered preliminary but warrant further research and replication.

## 5 Conclusion

We found that ongoing pain in chronic knee OA is characterised by increased brain activity in limbic-affective regions thus providing novel evidence for a strong emotional component of arthritis pain. This is further supported by the finding that limbic CBF-pain associations were largely accounted for by trait anxiety and, to a lesser degree, by pain catastrophizing. Taken together, in chronic OA pain, we demonstrate that only aMCC encodes pain intensity independently from negative affect whilst the involvement of emotional circuits is driven by trait anxiety.

## Conflict of interest statement

The authors have no conflicts of interest to declare.

The study was funded by Arthritis Research UK (Grant 18769).

## Figures and Tables

**Table 1:** Patient Demographics and group differences.

Data	Knee OA patients	Healthy controls	P-value
N.	26	27	–
Median age (range)	67.5 (54–84)	65.0 (57–80)	0.076
N. Males	12	9	0.35
Laterality of affected knee	12 left/14 right	–	–
N. Right-handed	24	23	–
Median educational scores	6^b^	3	0.023
VAS 0–100	40.2 (10–80)	–	–
PainDETECT^c^	12.5 (0–25)	–	–
BDI (range)^c^	7.8 (0–19)	2.5 (0 − 12)	0.0003
STAI-S	31.7 (20–55)^a^	26.4 (20–49)^a^	0.037
STAI-T	41.4 (21–70)^a^	30.7 (20–52)^a^	0.004
PCS	13.6 (1–34)	11.7 (0–29)	0.438
PCS: helplessness	5.6 (1–14)	4.2 (0 − 13)	0.203
PCS: magnification	2.4 (0–6)	2.3 (0–7)	0.903
PCS: rumination	5.6 (0–15)	5.2 (0 − 20)	0.749

Displayed are the mean (range) values unless elsewise specified. BDI – Beck's Depression Index, STAI-S – State Anxiety, STAI-T – Trait Anxiety, PCS – Pain Catastrophizing Scale.

a 1 subject score missing.

b 2 subjects were missing.

c Scores reported are the raw questionnaire scores.

**Table 2:** Areas with significant associations between CBF and reported ongoing OA pain intensity.

Anatomical regions	Cluster extent	X	Y	Z	T-score
Positive correlations					
L. Central Operculum	2983	− 42	0	12	5.05
L. Putamen		− 24	4	8	4.5
L. Brain Stem		− 4	− 34	− 4	4.76
L. Posterior Thalamus		− 10	− 32	6	4.24
L. Hippocampus		− 28	− 24	− 12	4.08
R. Hippocampus		28	− 26	− 10	3.77
L. Mid-Insula		− 40	− 2	2	3.43
L. Amygdala		− 22	− 6	− 12	3.62
R. Amygdala		18	− 10	− 14	3.24
aMCC	253	2	2	34	5.49
R. Subgenual ACC	79	4	26	− 6	5.04
L. Subgenual ACC		− 2	26	− 6	4.22
R. TOF	68	38	− 40	− 12	3.73

All results at FWE corrected P < 0.05, reported in MNI152 standard space.

L. – Left, R. – Right, aMCC – Anterior Mid-Cingulate Cortex, TOF – Temporal Occipital Fusiform.

**Table 3:** Areas with significant associations between CBF and reported ongoing OA pain intensity additionally controlled for pain catastrophizing and trait-anxiety scores.

Anatomical regions	Cluster extent	X	Y	Z	T-score
VAS × CBF controlled for STAI-T					
aMCC	11	− 4	6	32	5.84
aMCC	2	− 4	6	44	5.84

VAS × CBF controlled for PCS					
L. Putamen	1261	− 30	− 6	− 4	6.63
L. Amygdala		− 24	− 6	− 14	4.72
L. Hippocampus		− 22	− 16	− 16	3.82
L. Central Operculum		− 42	− 2	12	3.99
L. Insular Cortex		− 40	− 4	4	3.93
aMCC	331	− 2	8	38	7.4
R. Putamen	195	32	− 6	− 2	4.87
R. Amygdala		26	− 6	− 14	3.70
Brain Stem	100	0	− 20	− 22	4.14
PCC	19	− 2	− 20	46	4.83
R. Anterior Insular Cortex	17	28	14	− 14	4.38
R. Central Operculum	6	44	− 2	10	3.91

All results at FWE corrected P < 0.05, reported in MNI152 standard space. L. – Left, R. – Right, aMCC – Anterior Mid-Cingulate Cortex, S2 – Secondary Somatosensory Cortex, PCC – Posterior Cingulate Cortex, STAI-T – Trait Anxiety, PCS – Pain Catastrophizing Scale.

**Table 4:** Bivariate Pearson correlation scores between psychometric scores and CBF in regions of interest.

	BDI	PCS	aMCC	sgACC	L. Amyg	R. Amyg	L. Hipp	R. Hipp
Trait	0.359 (0.078)	0.649 (0.000)	0.171 (0.415)	0.120 (0.568)	0.169 (0.419)	0.225 (0.281)	0.169 (0.420)	0.083 (0.693)
BDI		0.359 (0.078)	0.234 (0.250)	0.293 (0.147)	0.336 (0.093)	0.335 (0.095)	0.327 (0.103)	0.312 (0.120)
PCS			0.239 (0.250)	0.390 (0.054)	0.073 (0.729)	0.245 (0.239)	0.317 (0.123)	0.298 (0.148)

All values are reported r-correlation values (P-value) from between variables carried out in SPSS 22. Abbreviations: L – Left, R – Right, Trait – STAI Trait anxiety score, BDI – Rasch-converted BDI-II scores, PCS – Pain Catastrophizing Scale, sgACC – subgenual Anterior Cingulate Cortex, Amyg – Amygdala, Hipp - Hippocampus.

## Appendix A Supplementary data

Supplementary material. Image 1
