# Supplementary material for: Associations of limbic-affective brain activity and severity of ongoing chronic arthritis pain are explained by trait anxiety
Source: Neuroimage Clin. 2016 Jul 1;12:269–76. doi: 10.1016/j.nicl.2016.06.022 (PMC4969259; doi:10.1016/j.nicl.2016.06.022)
Supplement: Supplementary file 1 — Supplementary material. [file mmc1.docx]

**Supplementary Material**

**for**

**Associations of limbic-affective brain activity and severity of ongoing chronic arthritis pain are explained by trait anxiety.**

Cottam, WJ., Condon, L., Alshuft, H., Reckziegel, D., Auer DP.

**Supplementary Figure 1.** Individual mean group CBF maps for A) OA patients and B) healthy subjects corrected for age, sex and mean CBF respectively (displayed in radiological convention).


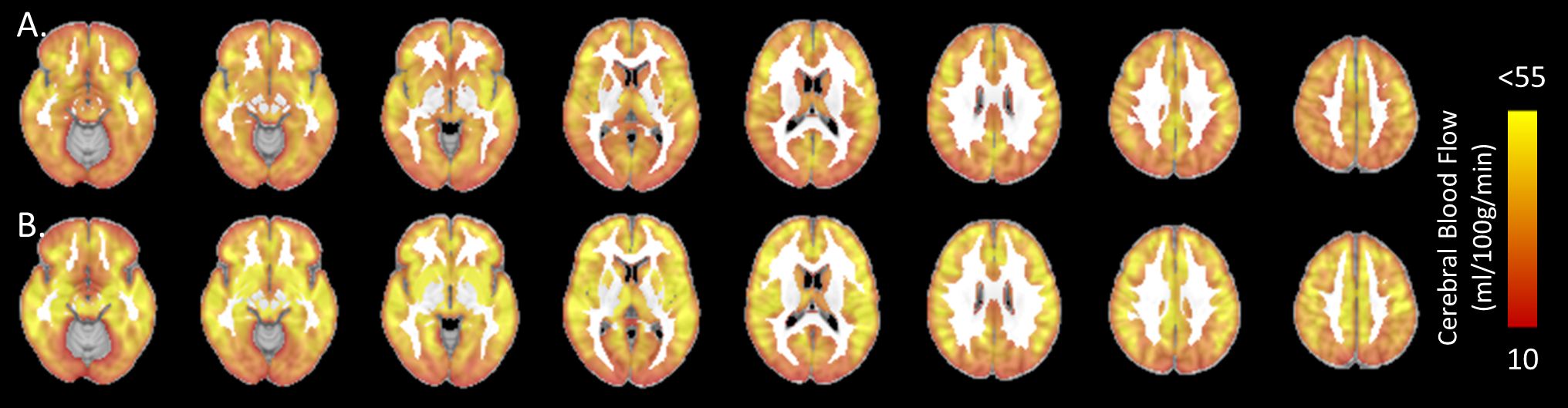


**Supplementary Figure 2.** Brain regions where CBF correlates positively (FWE p<0.05) with reported VAS scores before and after flipping all image data in those patients with osteoarthritis in their left knee (correcting for age, sex, and mean CBF). All images are shown in radiological format (right hemisphere is displayed on the left of the figure).


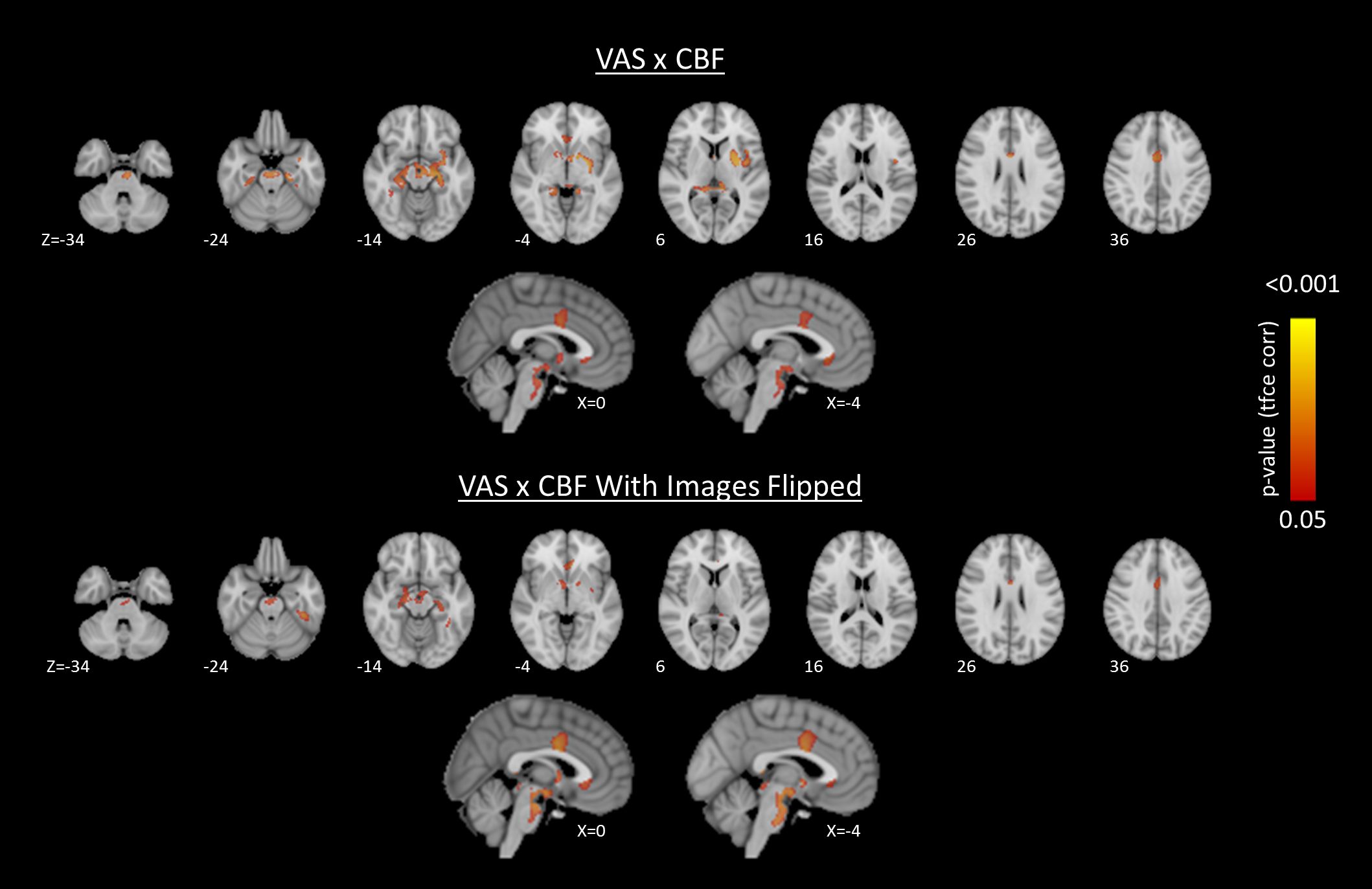


**Rasch conversion of BDI-II scores**

Rasch analysis of BDI-II reports from 180 subjects (age 39-90 years, 93 females) was carried out by Professor Nadina Lincoln and Dr Bryan Moreton on a separate study within the ARUK Pain Centre at the University Of Nottingham whom kindly made this data available.

Conversion was carried out by doing the following:

Rescore items 3 and 10 as follows: 0 = 0, 1 = 1, 2 = 1 and 3 = 2. Rescore items 9, 18 and 21 as follows: 0 = 0, 1 = 1, 2 = 1 and 3 = 1. Rescore item 16 as follows: 0 = 0, 1 = 1, 2 = 2 and 3 = 2. Discount scores from items 1, 5, 7 and 14. Sum the scores from the remaining items to produce a value from 0 to 42. Look up the score in the table below and match it to the corresponding ‘Rasch’ score:

| *Standard Score* | *‘Rasch’ Score* |
| --- | --- |
| 0 | -7.76 |
| 1 | -5.47 |
| 2 | -4.27 |
| 3 | -3.64 |
| 4 | -3.18 |
| 5 | -2.80 |
| 6 | -2.48 |
| 7 | -2.19 |
| 8 | -1.92 |
| 9 | -1.68 |
| 10 | -1.44 |
| 11 | -1.23 |
| 12 | -1.02 |
| 13 | -0.82 |
| 14 | -0.64 |
| 15 | -0.48 |
| 16 | -0.32 |
| 17 | -0.19 |
| 18 | -0.06 |
| 19 | 0.06 |
| 20 | 0.16 |
| 21 | 0.27 |
| 22 | 0.37 |
| 23 | 0.47 |
| 24 | 0.57 |
| 25 | 0.67 |
| 26 | 0.77 |
| 27 | 0.88 |
| 28 | 0.98 |
| 29 | 1.09 |
| 30 | 1.21 |
| 31 | 1.33 |
| 32 | 1.46 |
| 33 | 1.60 |
| 34 | 1.75 |
| 35 | 1.92 |
| 36 | 2.12 |
| 37 | 2.37 |
| 38 | 2.67 |
| 39 | 3.09 |
| 40 | 3.74 |
| 41 | 5.16 |
| 42 | 8.32 |

**Rasch conversion of PainDETECT scores**

Discount scores from the picture selection item (about the ‘course’ of pain) and the yes/no item (asking does pain ‘radiate’). Sum the scores from the remaining items to produce a value from 0 to 35. Look up the score in the table below and match it to the corresponding ‘Rasch’ score:

| *Standard Score* | *‘Rasch’ Score* |
| --- | --- |
| 0 | -2.89 |
| 1 | -2.22 |
| 2 | -1.80 |
| 3 | -1.53 |
| 4 | -1.34 |
| 5 | -1.18 |
| 6 | -1.04 |
| 7 | -0.92 |
| 8 | -0.81 |
| 9 | -0.71 |
| 10 | -0.62 |
| 11 | -0.53 |
| 12 | -0.45 |
| 13 | -0.38 |
| 14 | -0.30 |
| 15 | -0.23 |
| 16 | -0.16 |
| 17 | -0.09 |
| 18 | -0.02 |
| 19 | 0.06 |
| 20 | 0.13 |
| 21 | 0.21 |
| 22 | 0.29 |
| 23 | 0.37 |
| 24 | 0.46 |
| 25 | 0.55 |
| 26 | 0.65 |
| 27 | 0.76 |
| 28 | 0.88 |
| 29 | 1.02 |
| 30 | 1.18 |
| 31 | 1.39 |
| 32 | 1.65 |
| 33 | 2.01 |
| 34 | 2.56 |
| 35 | 3.42 |
